# Detrimental effects of specific Periodontopathic bacterial infection on tachyarrhythmia compared to Bradyarrhythmia

**DOI:** 10.1186/s12872-017-0703-2

**Published:** 2017-10-17

**Authors:** Norio Aoyama, Jun-ichi Suzuki, Naho Kobayashi, Tomoya Hanatani, Norihiko Ashigaki, Asuka Yoshida, Yuka Shiheido, Hiroki Sato, Hidetoshi Kumagai, Yuichi Ikeda, Hiroshi Akazawa, Issei Komuro, Masato Minabe, Yuichi Izumi, Mitsuaki Isobe

**Affiliations:** 1 Division of Periodontology, Department of Oral Interdisciplinary Medicine, Graduate School of Dentistry, Kanagawa Dental University, 82 Inaokacho, Yokosuka, Kanagawa 238-8580 Japan; 2 Department of Advanced Clinical Science and Therapeutics, The University of Tokyo, 7-3-1 Hongo, Bunkyo-ku, Tokyo, 113-8655 Japan; 3 Department of Cardiovascular Medicine, Tokyo Medical and Dental University, 1-5-45 Yushima, Bunkyo-ku, Tokyo, 113-8549 Japan; 4 Department of Periodontology, Graduate School of Medical and Dental Sciences, Tokyo Medical and Dental University, 1-5-45 Yushima, Bunkyo-ku, Tokyo, 113-8549 Japan; 5 Division of Periodontology, Kyushu Dental University, 2-6-1 Manazuru, Kokurakita-ku, Kitakyushu, Fukuoka, 803-8580 Japan; 6 Department of Oral Microbiology, Tsurumi University, 2-1-3 Tsurumi, Tsurumi-ku, Yokohama, Kanagawa 230-8501 Japan; 7 Department of Cardiovascular Medicine, The University of Tokyo, 7-3-1 Hongo, Bunkyo-ku, Tokyo, 113-8655 Japan

**Keywords:** Arrhythmia, Periodontal disease, Bacteria

## Abstract

**Background:**

Tachyarrhythmia (TA) and bradyarrhythmia (BA) are cardiac rhythm disorders that result in the decline of quality of life. While patients with periodontitis are at a high risk of cardiovascular disease (CVD), little causal information between TA and BA has been provided to date. To assess the relationship, periodontal bacterial infection in patients with TA or BA was evaluated.

**Methods:**

The subjects were patients with TA (*n* = 98) or BA (*n* = 40) who attended Tokyo Medical and Dental University hospital. Periodontal and blood examinations were performed. Periodontopathic bacterial existence in saliva was evaluated.

**Results:**

We found that specific periodontopathic bacteria, *Porphyromonas gingivalis* and *Prevotella intermedia*, were highly detected in saliva from TA patients compared to BA subjects. The rates of hypertension and dyslipidemia were comparable between the two groups.

**Conclusion:**

Specific periodontal bacterial infection might affect TA progression.

## Background

Periodontitis is an infectious oral disease that leads to destruction of the supporting tissues of teeth and finally induces loss of teeth. It is characterized as a chronic infection with periodontal bacteria. Many reports have shown that periodontal disease has a high prevalence around the world [1, 2]. Periodontitis is suspected as a possible risk factor for some systemic diseases including cardiovascular disease (CVD) [3]. Because CVD is an important cause of death, its prevention and treatment are significant health issues. Many studies indicated that patients with periodontal disease had a high risk for CVD events such as coronary artery disease (CAD), stroke and peripheral arterial disease [4–10].

Tachyarrhythmia (TA) and bradyarrhythmia (BA) are cardiac rhythm disorders that result in the decline of quality of life [11]. While patients with periodontitis are at a high risk of CVD, little causal information between arrhythmia and periodontitis has been provided to date. To assess the relationship, periodontal bacterial infection in patients with TA or BA were evaluated.

## Methods

### Study population

Subjects who were 71–90 years old were recruited from CVD patients with TA or BA in the Department of Cardiovascular Medicine in Tokyo Medical and Dental University Hospital between May 2012 and August 2015. One hundred and 38 subjects participated in this study and were subdivided into two age groups (71–80 year-old and 81–90 year-old). Excluded in this study were individuals who had a history and/or presence of other infectious diseases or did not consent to the participation. The Ethics Committees of the School of Medicine and the School of Dentistry, Tokyo Medical and Dental University approved the present study (744), and the protocol conformed to the Helsinki Declaration of 1975, as revised in 2013*.* Written informed consent was provided by the subjects.

### Medical examination

Medical doctors recorded medical and smoking history. TA and BA were diagnosed by electrocardiogram. Subjects who were diagnosed and/or treated as diabetes mellitus (DM), hypertension (HT) and dyslipidemia (DL) were recorded. Peripheral blood samples were obtained and level of C-reactive protein (CRP) was determined.

### Periodontal examination

Trained periodontists counted the number of residual teeth. Probing pocket depth (PPD), clinical attachment level (CAL) and bleeding on probing (BOP) at six points per tooth (buccal-mesial, mid-buccal, buccal-distal, lingual-mesial, mid-lingual and lingual-distal) on an upper right molar, an upper incisor, an upper left molar, a lower right molar, a lower incisor and a lower left molar were measured with a manual probe (PCP-UNC 15, Hu-Friedy, Chicago, IL, USA). When the representative tooth was missing, next tooth was used.

### Detection of bacteria

We obtained unstimulated saliva and used DNeasy Blood and Tissue kit (Qiagen, Tokyo, Japan) according to manufacturer’s instructions to extract bacterial DNA. Real-time polymerase chain reaction (PCR) was used to detect three major periodontal bacteria, *Porphyromonas gingivalis (P. gingivalis)*, *Aggregatibacter actinomycetemcomitans (A. actinomycetemcomitans)* and *Prevotella intermedia (P. intermedia)*. The real-time PCR was performed and specific primers for each bacterium were used as previously described [12].

### Statistical analysis

Numerical data was presented as means ± standard deviation (SD). Student’s *t*-test was used to compare numerical values such as age, CRP, teeth number, PPD, CAL and BOP. Chi-square test was performed to compare dichotomous variables such as sex, smoker rate, positive rates of DM, HT and DL and bacterial detection rates. Wilcoxon test was used to compare bacterial counts, because bacterial counts were not normally distributed. We used JMP 9.0.3 (SAS Institute Inc., Cary, NC, USA) for all statistical analyses and considered values of *p* < 0.05 significant.

## Results

### Characteristics of the patients

The characteristics of the subjects in the present study are shown in Table 1. There was no statistical difference of age, sex, smoking rate, HT, DL and CRP between the groups. Prevalence of DM was higher in the TA group compared with the BA group in 81–90 year-old patients, but not in 71–80 year-old patients.

**Table 1:** Characteristics of the subjects

Group	Bradyarrhythmia	Tachyarrhythmia
(A) 71–80 year-old patients		
Number	30	79
Age	75.1+/−2.4	74.3+/−2.4
Sex (Female %)	23	27
Smoking [%]	37	43
Diabetes [%]	47	32
Hypertension [%]	77	71
Dyslipidemia [%]	50	38
CRP [mg/dL]	0.38+/−0.56	0.36+/−0.93
(B) 81–90 year-old patients		
Number	10	19
Age	83.5+/−2.9	83.2+/−2.2
Sex (Female %)	30	42
Smoking [%]	10	32
Diabetes [%]	0	26*
Hypertension [%]	70	89
Dyslipidemia [%]	20	42
CRP [mg/dL]	0.79+/−2.12	1.20+/−3.21

*p < 0.05 between the groups

### Periodontal conditions

The number of missing teeth was comparable between the two arrhythmia groups of the same age categories (Table 2). There was no statistical difference of mean PPD, CAL and BOP rate between the groups.

**Table 2:** Periodontal condition

Group	Bradyarrhythmia	Tachyarrhythmia
(A) 71–80 year-old patients		
Number of missing teeth	12.9 +/− 9.2	14.4 +/− 9.5
PPD [mm]	2.44 +/− 0.52	2.56 +/− 0.68
CAL [mm]	3.10 +/− 1.11	3.57 +/− 1.20
BOP [%]	16 +/− 22	22 +/− 24
(B) 81–90 year-old patients		
Number of missing teeth	17.1 +/− 9.7	16.7 +/− 8.4
PPD [mm]	2.41 +/− 0.30	2.39 +/− 0.55
CAL [mm]	3.65 +/− 1.09	3.34 +/− 1.06
BOP [%]	17 +/− 17	12 +/− 20

### Bacterial counts

The counts of *P. intermedia* in the TA group increased in comparison to the BA group in 71–80 year-old patients (Table 3). The TA group also had an increased amount of *P. gingivalis* compared to the BA group in 81–90 year-old patients (Table 3). The counts of *A. actinomycetemcomitans* were comparable between the two groups. We also showed a positive rate of *P. intermedia* (Table 4). The TA group had an increased detection rate of compared to the BA group in 71–80 year-old patients.

**Table 3:** Bacterial counts in saliva

	Bradyarrhythmia	Tachyarrhythmia	*P*
(A) 71–80 year-old patients			
*P. gingivalis*	5.5 × 10^3^ (0, 4.8 × 10^5^)	1.4 × 10^5^ (3.0 × 10^2^, 3.7 × 10^6^)	0.1118
*A. actinomycetemcomitans*	0 (0, 0)	0 (0, 0)	0.8187
*P. intermedia*	0 (0, 0)	0 (0, 8.4 × 10^2^)	0.0193*
(B) 81–90 year-old patients			
*P. gingivalis*	6.7 × 10^2^ (1.4 × 10^2^, 4.1 × 10^4^)	6.8 × 10^6^ (3.7 × 10^4^, 2.3 × 10^7^)	0.0062*
*A. actinomycetemcomitans*	0 (0, 0)	0 (0, 1.1 × 10^2^)	0.5630
*P. intermedia*	0 (0, 0)	0 (0, 7.0 × 10^3^)	0.1267

Values are shown as median [counts/mL] (first and third quartile)

*p < 0.05 between the groups

**Table 4:** Positive rate of bacteria in saliva

	Bradyarrhythmia	Tachyarrhythmia	*P*
(A) 71–80 year-old patients			
*P. gingivalis*	59%	80%	0.0581
*A. actinomycetemcomitans*	18%	18%	0.9473
*P. intermedia*	9%	35%	0.0105*
(B) 81–90 year-old patients			
*P. gingivalis*	78%	100%	0.0713
*A. actinomycetemcomitans*	11%	20%	0.5921
*P. intermedia*	11%	40%	0.1415

*p < 0.05 between the groups

## Discussion

In this study, we found that specific periodontopathic bacteria, *P. gingivalis* and *P. intermedia*, were highly detected in the TA subjects compared to the BA subjects.

### Periodontitis affects arrhythmia

It is well known that there is a relationship between periodontitis and CVD. However, there are few papers to show the association between arrhythmia and periodontitis. It is well recognized that ischemic stroke is frequently induced by TA. Clinically, a recent case-control study showed that high levels of periodontal clinical attachment loss, gingivitis and radiographic bone loss were independently associated with stroke, while the etiology of the stroke was not indicated [13]. On the other hand, Holm-Pedersen et al. demonstrated that there might be a link between active root caries and cardiac arrhythmias in elderly persons, while there was no association between periodontal disease and arrhythmia in the study [14]. In an animal model, Yu et al. showed the effect of periodontitis on susceptibility to atrial fibrillation, which is a major TA. Periodontitis was induced by tying 2–0 silk ligatures at the second premolar of mandibula in adult mongrel canines. Electrophysiologic evaluation was performed to assess atrial refractoriness and atrial fibrillation inducibility. They found that periodontitis induced inflammatory responses in atrial myocardium, which disturbed the structural and electrophysiologic properties of the atrium and facilitated atrial fibrillation [15]. Therefore, periodontitis may deteriorate TA such as atrial fibrillation.

### Specific Periodontopathic bacteria infection may deteriorate TA

There are a limited number of reports to evaluate the influence of systemic periodontal pathogen infections on TA and/or stroke. Hosomi et al. demonstrated a relationship between serum antibodies against periodontal pathogens and ischemic stroke. They evaluated patients with acute ischemic stroke and patients without previous stroke or stroke subtype. The results showed that the serum-antibody level of *P. intermedia* was significantly higher in athero-thrombotic stroke patients than in patients with no previous stroke. Serum antibody against *P. gingivalis* was also significantly associated with atrial fibrillation. They concluded that infection with specific periodontal bacteria was associated with stroke and atrial fibrillation [16]. Pussinen et al. also showed serological evidence that a chronic infection caused by *P. gingivalis* and *A. actinomycetemcomitans* was associated with stroke incidence in prospective case-control studies [17, 18], while the etiology of the stroke was not clarified. These previous studies suggested that periodontal pathogens such as *P. gingivalis* and *P. intermedia* may have some crucial effects on stroke and/or TA. The mechanisms connecting periodontal infection and TA are not clear, but long-term systemic exposure to periodontal pathogens may influence cardiac homeostasis.

## Conclusions

In the present study, we revealed that specific periodontopathic bacteria, *P. gingivalis* and *P. intermedia*, were highly detected in subjects with TA compared to BA. Thus, we can conclude that specific periodontopathic bacterial infection may affect TA development. Generally, the elderly cardiovascular patients including arrhythmia patients have a higher prevalence of asymptomatic disorders than young patients. And also the elderly patients often have other systemic diseases. These factors may reduce statistical sensitivity of symptomatic arrhythmia. This may be a reason why elderly people positive for periodontal bacteria had decreased symptoms of TA or BA. Recently, we demonstrated that *P. gingivalis* influenced myocardial remodeling after ischemia [19]. Another study showed that *P. intermedia* might have some effects on cardiovascular disease in Marfan syndrome patients [20]. Thus, these two specific bacteria may affect TA through myocardial remodeling in clinical settings. Further investigation is needed to reveal the detailed causal relationship between arrhythmia and specific periodontopathic bacterial infection.

## Abbreviation

*A. actinomycetemcomitans*: *Aggregatibacter actinomycetemcomitans*
BA: Bradyarrhythmia
BOP: Bleeding on probing
CAD: Coronary artery disease
CAL: Clinical attachment level
CRP: C-reactive protein
CVD: Cardiovascular disease
DL: Dyslipidemia
DM: Diabetes mellitus
HT: Hypertension
*P. gingivalis*: *Porphyromonas gingivalis*
*P. intermedia*: *Prevotella intermedia*
PCR: Polymerase chain reaction
PPD: Probing pocket depth
SD: Standard deviation
TA: Tachyarrhythmia
